# Genesis According to Science (so Called)

**Published:** 1875-05-01

**Authors:** 


### GENESIS ACCORDING TO SCIENCE (SO CALLED).

*From the Canada Lancet.*

WE cannot resist giving our readers the amusement — though the feeling excited will probably be by no means one of unmixed amusement—of reading the following smart concrete statement of some modern "scientific" schemes of creation from one of our Trans-Atlantic contemporaries:

### THE NEW SCRIPTURES ACCORDING TO TYNDAL AND OTHERS.

1. Primarily the Unknowable moved upon cosmos and evolved protoplasm.

2. And protoplasm was inorganic and undifferentiated, containing all things in potential energy; and a spirit of evolution moved upon the fluid mass.

3. And the Unknowable said, Let atoms attract; and their contact begat light, heat and electricity.

4. And the Unconditioned differentiated the atoms, after its kind; and their combinations begat rock, air and water.

5. And there went out a spirit of evolution from the Unconditioned; and, working in protoplasm by accretion and absorption, produced the organic cell.

6. And cell, by nutrition, evolved primordial germ, and germ developed protogene, and protogene begat eozoon, and eozoon begat monad, and the monad begat animalcule.

7. And animalcule begat ephemera; then began creeping things to multiply on the face of the earth.

8. And earthy atom in vegetable protoplasm begat the molecule; and thence came all grass and every herb on the earth.

9. And the animalcule in the water evolved fins, tails, claws, and scales; and in the air wings and beaks; and on the land they sprouted such organs as were necessary as played upon by the environment.

10. And by accretion and absorption, came the radiata and mollusca; and mollusca begat articulata, and articulata begat vertebrata.

11. Now these are the generations of the higher vertebrata, in the cosmic period that the Unknowable evoluted the bipedal mammalia.

12. And every man of the earth, while he was yet a monkey, and the horse, while he was a hipparion, and the hipparion before he was an oredon.

13. Out of the ascidian came the amphibian and begat the pentadactyle; and the pentadactyle by inheritance and selection produced the hylobate, from which are the simiadæ in all their tribes.

14. And out of the simiadæ, the lemur prevailed above his fellows and produced the platyrhine monkey.

15. And the platyrhine begat the catarrhine, and the catarrhine monkey begat the anthropoid ape, and the ape begat the longimanous ourang, and the ourang begat the chimpanzee, and the chimpanzee evoluted the what-is-it.

16. And the what-is-it went into the land of Nod, and took him a wife of the longimanous gibbons.

17. And in process of the cosmic period, were born unto them and their children, the anthropomorphic primordial types.

18. The homunculus, the prognathus, the troglodyte, the autochthon, the terragen—these are the generations of primeval man.

19. And the primeval man was naked and not ashamed, but lived in quadrumanous innocence, and struggled mightily to harmonize with the environment.

20. And by inheritance and natural selection, did he progress from the stable and homogeneous, to the complex and heterogeneous; for the weakest died and the strongest grew and multiplied.

21. And man grew a thumb, for that he had need of it, and developed capacities for prey.

22. For, behold, the swiftest men caught the most animals, and the swiftest animals got away from the most men; wherefore, the slow animals were eaten, and the slow men starved to death.

23. And as the types were differentiated, the weaker types continually disappeared.

24. And the earth was filled with violence, for man strove with man, and tribe with tribe, whereby they killed off the weak and foolish, and secured the survival of the fittest.

